# Interferon-inducible CXC-chemokines are crucial immune modulators and survival predictors in colorectal cancer

**DOI:** 10.18632/oncotarget.21286

**Published:** 2017-09-28

**Authors:** Larissa Kistner, Dietrich Doll, Anne Holtorf, Ulrich Nitsche, Klaus-Peter Janssen

**Affiliations:** ^1^ Department of Surgery, Klinikum rechts der Isar, TUM, Munich, Germany; ^2^ Current/Present Address: St. Marienhospital Vechta, Vechta, Germany

**Keywords:** colorectal cancer, tumor immunology, mouse model, chemokines

## Abstract

Tumor-infiltrating T-cells are strongly associated with prognosis in colorectal cancer, but the mechanisms governing intratumoral lymphocyte recruitment are unclear. We investigated the clinical relevance and functional contribution of interferon-regulated CXC-chemokines CXCL9, CXCL10, and CXCL11, described as T-cells attractants. Their expression was significantly elevated in tumors as compared to normal colon in 163 patients with colon cancer, represented an independent positive predictor of post-operative survival, and was highly significantly correlated with the presence of tumor-infiltrating cytotoxic CD8^+^ T-cells and CD4^+^ T_H1_-effector cells. The regulation of chemokine expression was investigated in established cell lines and in tissue explants from resected tumor specimen (n=22). All colorectal cancer cell lines tested, as well as stroma or endothelial cells, produced CXC-chemokines in response to cytokine stimulation. Moreover, resected tumor explants could be stimulated to produce CXC-chemokines, even in cases with initially low CXC-levels. Lastly, a causative role of chemokine expression was evaluated with an orthotopic mouse model, based on isogenic rectal CT26 cancer cells, engineered to express CXCL10. The orthotopic model demonstrated a protective and anti-metastatic role of intratumoral CXCL10 expression, mediated mainly by adaptive immunity.

## INTRODUCTION

Cancer of colon and rectum is amongst the most common malignancies [1]. However, the current tumor staging system is not well suited for individualized risk assessment [2]. As potential biomarkers for personalized prognosis, we recently proposed a group of chemokines (CXCL9, CXCL10, and CXCL11), as part of a 19-gene expression signature [3, 4]. High intratumoral expression of the three chemokines, and of Granzyme B, a marker for cytotoxic T-cells and NK-cells, was indicative of good prognosis [4]. CXC-chemokine signaling regulates angiogenesis and recruitment of immune cells [5], it connects cancer cells and the surrounding stroma [6]. CXCL9 (MIG), CXCL10 (IP10) and CXCL11 (ITAC) are IFN_γ_ inducible chemokines of the CXC-family [7, 8]. These chemokines have angiostatic function and are pivotal for the recruitment and activation of leukocytes, mediated by binding to receptor CXCR3, preferentially expressed on activated T cells [9]. Recently, CXCL11 expression was associated with good prognosis in the Cancer Genome Atlas [10]. Thus, the hypothesis could be raised that high intratumoral expression of CXCR3-ligands inhibits angiogenesis and induces infiltration of activated T cells. Colorectal tumors frequently contain prominent immune infiltrates, and mouse experiments support an anti-tumoral role of adaptive immunity [11, 12]. Moreover, prognosis in colorectal cancer is strongly correlated to tumor-infiltrating T cells, notably T_H1_ and CD8^+^ effector T cells [13–16]. A T-cell mediated immune response is able to inhibit carcinogenesis [17], also evidenced by mouse models [11, 12, 18]. In fact, the number and distribution of intratumoral T-cells outperforms the established TNM staging system in terms of prognostic power [16, 19]. However, the mechanism underlying T-cell infiltration into solid tumors is not well understood, and the contribution of interfon-regulated CXC-chemokines is under debate [20]. Therefore, we evaluated the contribution of the CXCR3-ligands CXCL9, CXCL10 and CXCL11 to colorectal carcinogenesis by analysis of their expression and prognostic relevance in human colorectal cancer tissue. Further, we analyzed the regulation of their expression in primary and established colon cancer cell lines, as well as in non-cancer cells from the tumor stroma. Furthermore, expression of the CXCR3-ligands was investigated in genetic mouse models for digestive cancer, except for CXCL11, which is not expressed in the standard genetic mouse background C57Bl/6. Further, a causal *in vivo* role of CXCR3-ligands was assessed with the help of an orthotopic colon cancer model.

## RESULTS

### Differential expression of interferon regulated CXC-chemokines in colon cancer

**Figure 1:**
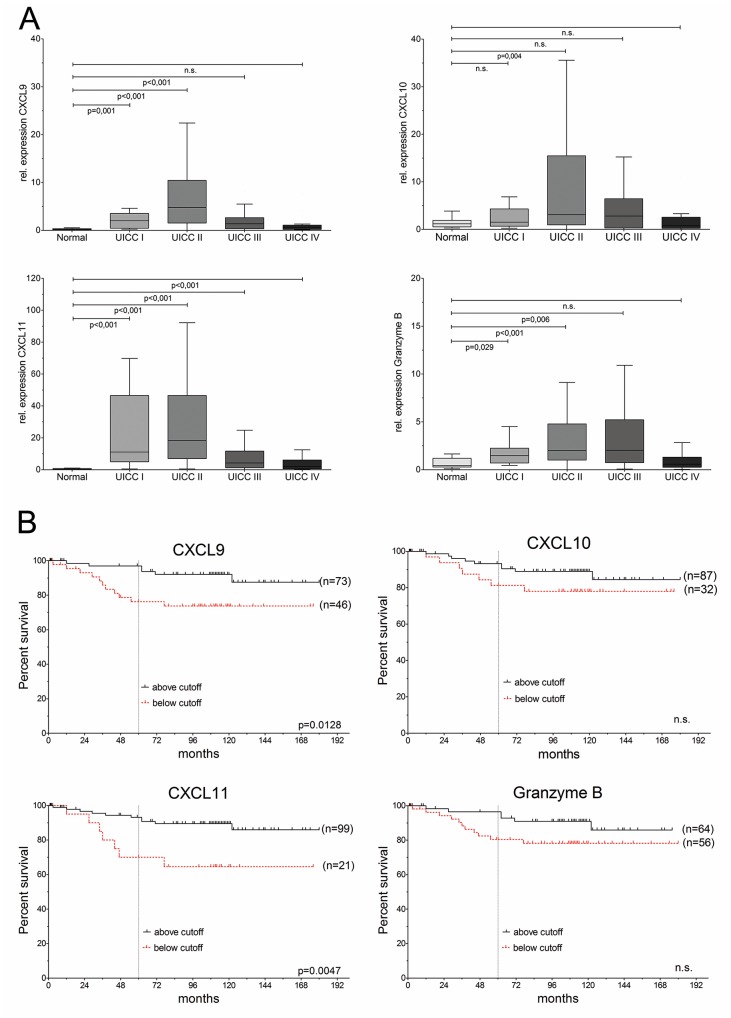
CXC-chemokines are differentially expressed and associated with good prognosis in colorectal cancer **(A)** Expression of CXCL9, CXCL10, CXCL11 and Granzyme B in UICC/AJCC stages I (n=13), II (n=75), III (n=36), IV (n=39), compared to normal colon (n=28). **(B)** Kaplan-Meier analysis of overall survival (OS), based on cutoff values for expression.

We previously identified chemokines CXCL9, CXCL10, and CXCL11, as well as GZMB (Granzyme B), as part of a prognostic gene signature in colon cancer. Here, we validated the transcriptome findings on an independent patient collective by quantitative real-time-PCR (qRT-PCR), confirming their up-regulated expression in a patient collective with colorectal carcinoma, representing all stages of the disease (n=163 cases; clinical data summarized in Supplementary Table 1), compared to normal colon mucosa from 28 patients (Figure 1A). Pronounced differences were observed for CXCL9, which highly significantly up-regulated in all tumor stages (p<0.0001, all tumors vs. normal tissue), followed by CXCL11 (p<0.0001, all tumors vs. normal tissue) and Granzyme B (p<0.0003, all tumors vs. normal tissue), whereas CXCL10 showed significant upregulation in stage II, but a modest increase upon comparison of all tumor stages to normal colon (p=0.095). Furthermore, CXCL10 and CXCL11, but not CXCL9, were up-regulated in benign precursor lesions (Supplementary Figure 1A). A strong degree of co-expression was found in individual patients for all three chemokines and GZMB (Supplementary Figure 1B, Supplementary Table 2). Of note, CXCL10 and CXCL11 expression was significantly reduced in matched samples from colorectal liver metastasis as compared to primary cancer (n=11)(Supplementary Figure 1C).

### CXC-chemokine expression is associated with good prognosis

To avoid any bias, only patients with complete tumor resection (R0) were included in the following prognostic analysis (n=120, clinical data summarized in Supplementary Table 3). Cut-point analysis by maximally selected log-rank statistics yielded threshold values for each chemokine and Granzyme B, based on stratification for cancer-specific survival (Supplementary Figure 2A). Kaplan-Meier analysis was carried out based on these thresholds (Figure 1B). Expression of CXCL11 allowed the most stringent prediction of overall survival (HR: 3.7, 95%CI 1.4–9.8, p<0.0047), and disease-free survival (p=0.0042; Supplementary Figure 2B).

Univariable ‘time-to-event’ analysis showed that patients with high expression of CXCL9 or CXCL11 ad significantly increased cause-specific post-operative survival (CXCL9: hazard ratio (HR)=3.3, 95% CI: 1.3–8.9, p=0.019; CXCL11: hazard ratio (HR)=3.7, 95% CI: 1.4–9.8, p=0.008) (Table 1). The independence of prognostic ability of CXCL11-based recurrence risk stratification (and to a lesser extent, for CXCL9), was further evaluated and confirmed by multivariable analyses (Table 1). Hazard ratio estimates for CXCL11-based stratification remained essentially unchanged and retained significance after consecutive pair-wise adjustment for the most important clinical-pathological variables, which are currently used for risk evaluation in colorectal cancer: tumor staging (UICC/AJCC, based on pTNM categories), poor histological differentiation (tumor grading), lymphatic invasion, as well as age and sex of the patients as further putative confounding variables (Table 1). Of note, CXCL11-based risk stratification remained independent of all potential confounders upon pairwise comparison.

**Table 1:** Consecutive (one-by-one) adjustment for confounding factors

	Consecutive multivariable analysis (pairwise comparison)					
	Univariate analysis	Tumor stage (UICC/AJCC)(I/II vs. III/IV)	Histological grading(1/2 vs. 3/4)	Lymphatic invasion(yes/no)	Age (years)	Sex (female/male)
**CXCL9**	**p=0.019HR=3.30(95%CI 1.22-8.95)**	p=0.076 (n.s.)HR=2.60(95%CI 0.91-7.48)	**p=0.011HR=3.67(95%CI 1.34-10.0)**	**p=0.035HR=2.98(95%CI 1.08-8.23)**	**p=0.020HR=3.280(95%CI 1.21-8.90)**	**p=0.021HR=3.27(95%CI 1.20-8.92)**
**CXCL10**	p=0.193 (n.s.)HR=1.93(95%CI 0.718-5.18)	p=0.499 (n.s.)HR=1.43(95%CI 0.51-4.00)	p=0.100 (n.s.)HR=2.34(95%CI 0.85-6.42)	p=0.306 (n.s.)HR=1.69(95%CI 0.62-4.62)	p=0.219 (n.s.)HR=1.86(95%CI 0.69-5.01)	p=0.151 (n.s.)HR=2.07(95%CI 0.77-5.57)
**CXCL11**	**p=0.008HR=3.69(95%CI 1.40-9.76)**	**p=0.027HR=3.08(95%CI 1.13-8.34)**	**p=0.004HR=4.38(95%CI 1.62-11.8)**	**p=0.029HR=3.16(95%CI 1.13-8.85)**	**p=0.009HR=3.66(95%CI 1.39-9.73)**	**p=0.038HR=2.82(95%CI 1.06-7.49)**
**Granzyme B**	p=0.105 (n.s.)HR=3.40(95%CI 0.77-14.9)	p=0.128 (n.s.)HR=3.16(95%CI 0.72-13.9)	p=0.070 (n.s.)HR=3.96(95%CI 0.90-17.6)	p=0.087 (n.s.)HR=3.64(95%CI 0.83-16.0)	p=0.095 (n.s.)HR=3.53(95%CI 0.80-15.6)	p=0.120 (n.s.)HR=3.27(95%CI 0.73-14.5)

Moreover, CXCL11 expression allowed risk-stratification even in the clinically relevant subgroup of locally restricted colon cancer (UICC/AJCC stage II, n=71). Disease relapse by distant metastasis was significantly less frequent in stage II patients with above-threshold CXCL11 expression (16% recurrence rate), compared to the low-expressing group (50% recurrence, p=0.0431; Supplementary Table 4).

### Cancer cells and stroma contribute to CXC-chemokine production

**Figure 2:**
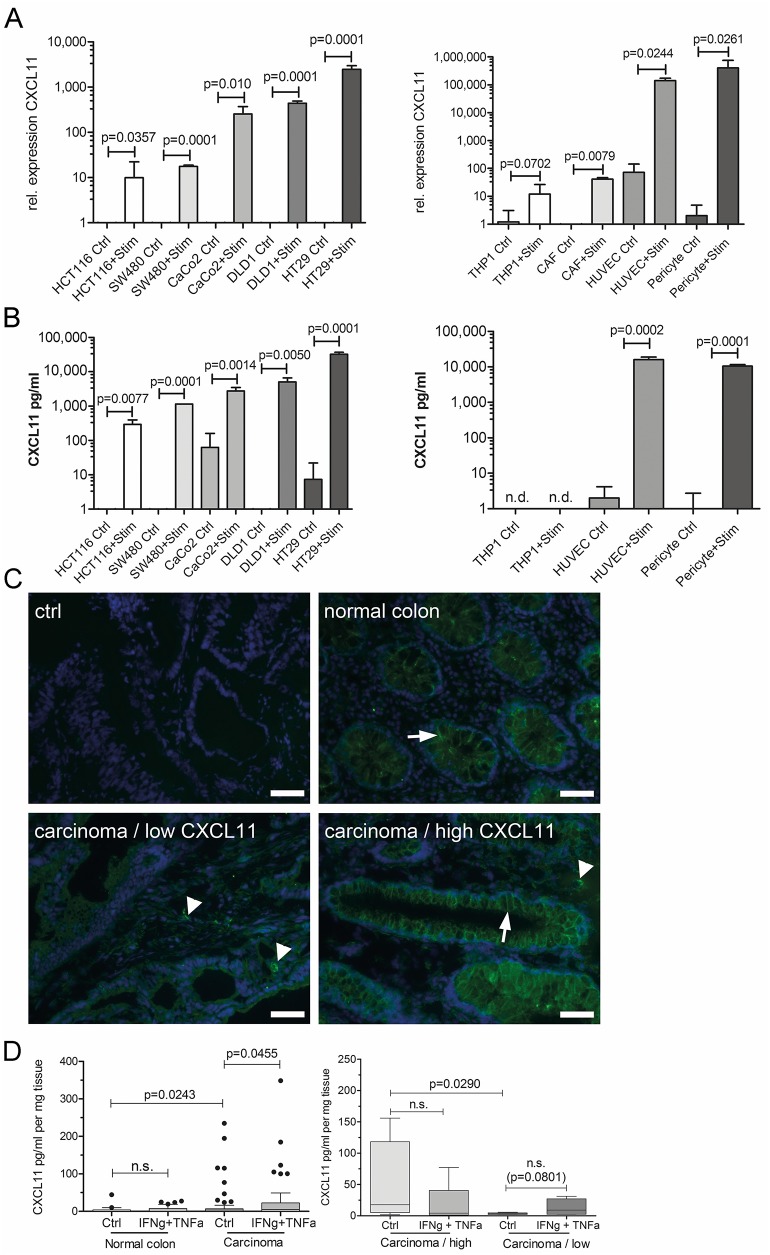
Cancer and stroma cells produce CXC-chemokines **(A)** Relative mRNA expression of CXCL11 in colorectal cancer cells (left side) or stroma cells (right side) under control conditions (ctrl), or in response to stimulation with TNFα+IFN _γ_ (Stim). **(B)** CXCL11 secretion by ELISA in colorectal cancer cells (left) or stroma cells (right). Values presented as mean±SD. **(C)** Detection of CXCL11 by specific staining on frozen sections. *Ctrl*: secondary antibody only; *normal colon*: note the staining of basolateral membrane of epithelia (arrow); *carcinoma/low CXCL11*: case with low CXCL11 mRNA expression; *carcinoma/high CXCL11*: case with above-threshold CXCL11 mRNA expression. Arrow denotes staining in carcinoma cells, arrowheads: stroma cells. Magnification 400x, sizebar: 20μm. **(D)** CXCL11 ELISA on supernatants after *ex vivo* culture of tumor samples and normal mucosa (n=22 patients). Left panel: CXCL11 secretion was significantly higher in carcinoma as compared to normal mucosa (ctrl). Cytokine stimulation lead to significantly increased CXCL11 expression in tumors, but not in normal colon. Right panel: stratification according to CXCL11 mRNA expression. Chemokine secretion was significantly increased in the group with “high CXCL11” expression (n=18), as compared to the “low CXCL11” group (n=7). Tumors from the initially low CXCL11 expressing group could be stimulated to secrete CXCL11, though not attaining significance.

**Figure 3:**
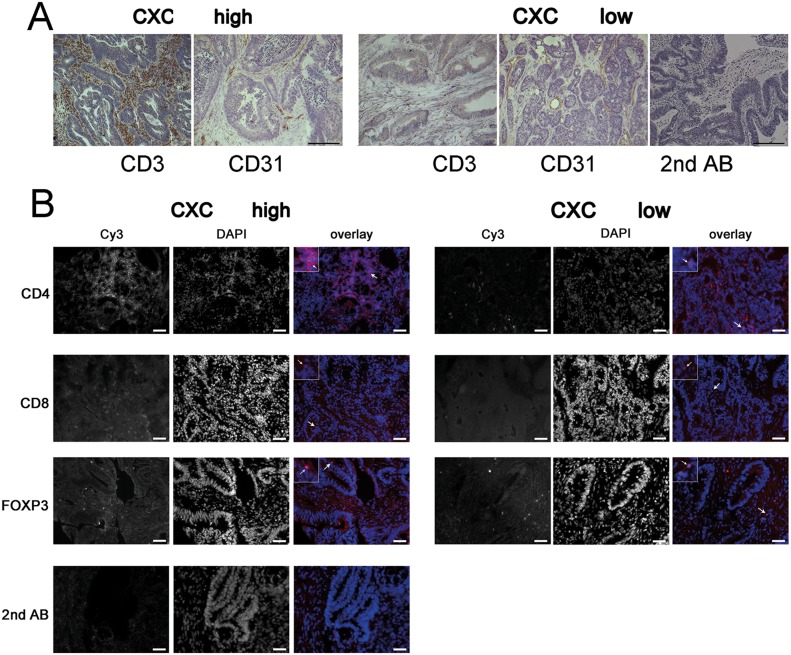
CXCL11 expression is correlated with T-cell infiltration **(A)** Representative immunohistochemistry staining of tissue samples from the highest (n=14) vs. lowest quartiles (n=12) of CXCL11 expression. T-cell density (CD3+), but not blood vessel density (CD31 staining), differs between both group. Control staining with secondary Ab only; size bar 20 μm. **(B)** Representative immunofluorescence staining for CD4, CD8, FoxP3, and secondary Ab only (shown in red), nuclear counterstaining (blue). Arrows denote staining for CD4 and CD8, and nuclear staining for FoxP3. Density of CD4^+^ and CD8^+^, but not FoxP3^+^ cells, is increased in tumors with high CXCL11 expression. Sizebar: 20 μm.

All colorectal cancer cell lines tested produced CXCL11 after stimulation with the cytokines IFN_γ_ and TNFα, as evidenced by qRT-PCR and by ELISA (Figures 2 and 3). Similar results were obtained for CXCL9 and CXCL10 (Supplementary Figure 3). However, the microsatellite unstable cell line HCT116 did not show significant upregulation of CXCL9 and CXCL10 after stimulation (Supplementary Figure 3). In addition, stroma cells were tested for CXCL11 expression. Human primary endothelial cells (HUVEC) and pericytes showed high CXCL11 expression and secretion after cytokine stimulation. Cancer-associated fibroblasts (CAF) derived from colorectal cancer, as well as monocyte-derived THP1 cells showed inducible CXCL11 expression on mRNA, but not on protein level (Figure 2). Next, CXCL11 expression was investigated in clinical samples by immunocytochemistry (n=21 patients, Figure 2C). Staining of tissue sections confirmed CXCL11 expression in cancer cells which were identified by glandular morphology and anti-EpCam staining (not shown). CXCL11 immunoreactivity was mainly detected at the basolateral surface of tumor cells, but reactivity was also observable in the stroma, in accordance with *in vitro* results (Figure 2B). Tumors with above-threshold CXCL11 mRNA expression (n=11) were strongly positive for CXCL11 protein, whereas low mRNA expressing tumors (n=10) showed no or weak signals on protein level (Fisher's exact test, p=0.0286).

### Tumor explants secrete CXC-chemokines

We analyzed fresh explants from tumor tissue and adjacent non-diseased mucosa prospectively from n=22 patients (clinical data summarized in Supplementary Table 5) for CXC-chemokine mRNA expression and protein secretion. Moreover, chemokine production was tested after cytokine stimulation *ex vivo*. Tumor tissue produced significantly more CXCL11 than normal mucosa, even without stimulation (p=0.0243; Figure 2D), whereas no significant differences were observed for CXCL10 (Supplementary Figure 4). Cytokine stimulation of carcinoma samples, but not of normal tissue, resulted in a significant increase of CXCL11 production (p=0.0455; Figure 2D, left panel). Next, we assigned the patients to high/low CXC-chemokine expressing groups, based on initial intratumoral CXC-chemokine mRNA expression. Importantly, ELISA results were in good accordance with transcript levels, and CXCL11 protein secretion was significantly higher in tumors with above-threshold CXCL11 mRNA expression (“high”, n=17) compared to “low” expressing tumors (n=5; p=0.0290; Figure 2D, right panel). In samples with initially low CXCL11 expression, increased secretion of CXCL11 could be achieved by cytokine stimulation, even though the difference did not attain significance (p=0.0801, Figure 2D, right panel). Samples with initially “high” CXCL11 expression could not be further induced to produce more CXCL11 after cytokine treatment, rather showing a trend to decreased CXCL11 production.

### Density of intratumoral T-cells is associated with chemokine expression

**Figure 4:**
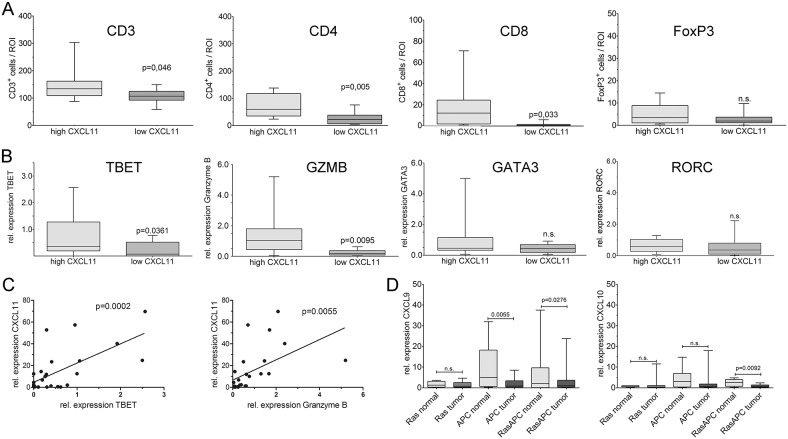
CXCL11 expression is correlated with T_H1_-type infiltration Blinded quantification of patient samples from the highest (n=14) vs. lowest quartiles (n=12) of chemokine expression. **(A)** Significantly more CD3^+^ and CD8^+^ cells, and highly significantly more CD4^+^ cells are found in tumors with high CXCL11 expression. No significant differences were observable for FoxP3^+^ cells. **(B)** Quantification of T-cell transcripts in CXCL11 high vs. low tumors by qPCR. Expression of TBET (T_H1_) and GZMB (CTL) significantly differed between both groups, whereas no differences were observed for GATA3 (T_H2_) and RORC (T_H17_). **(C)** Expression of TBET and GZMB, respectively, is positively correlated to CXCL11. **(D)** Chemokine expression is negatively correlated with tumor aggressiveness in mouse models. MurineCXCL9 and CXCL10 transcripts were quantified by qPCR in intestinal tumors and normal mucosa (n=6 mice/group); C57Bl/6 mice are naturally CXCL11-deficient. Chemokine expression is significantly reduced in the compound transgenic RasApc model with aggressive tumor formation, but not in single transgenic Ras-mice (pvillin-Kras^V12G^), which mainly display benign lesions. Apc mutated mice (Apc^1638N^) show intermediate behaviour.

CXCL9, CXCL10 and CXCL11 are known as T-cell chemoattracting cytokines. Thus, samples from the highest (n=14) and lowest (n=12) quartile of CXCL11 expression were analyzed by immunostaining and qRT-PCR for T-cell infiltrates. Both patient groups had essentially the same age, sex, and tumor stage distribution. Two observers, blind to sample identity, analyzed the number of immune cells in ten high-power regions from the central areas of tumors. Patients with above-threshold CXC expression had significantly higher numbers of CD3^+^ T-cells (p=0.046), highly significantly increased CD4^+^ T-helper cells (p=0.005), and significantly more CD8^+^ cytotoxic T-cells (p=0.033) (Figure 4A). There were no significant differences in the density of FoxP3^+^ regulatory T-cells (p=0.141). L-selectin (CD62L), an adhesion molecule expressed in naïve T-cells, was indistinguishable between both patient groups (p=0.244) and stained few cells, indicating that the majority of intratumoral T-lymphocytes are activated (Supplementary Figure 5). In order to confirm and expand the immunostaining results, T-cell specific transcripts were analyzed by qRT-PCR. The group with highest CXC levels showed highly significantly increased expression of GZMB (p=0.0095), and significantly higher levels of TBET (T-box transcription factor 21; p=0.0361), a hallmark transcription factor of T_H1_ cells (Figure 4B), as compared to cases with low CXC expression. No significant differences were found for the T_H2_-type transcription factor GATA3 (GATA binding protein 3), the T_H17_-type transcription factor RORC (ROR_γ_t, RAR-related orphan receptor C) (Figure 4B), and the regulatory T-cell marker FOXP3 (Supplementary Figure 5). Regression analysis confirmed a highly significant correlation between expression of CXCL11 and TBET (p=0.0002), or Granzyme B (p=0.0055), respectively (Figure 4C), but not between CXCL11 and GATA3, RORC or FOXP3 (not shown).

Next, blood vessel density was investigated with the endothelial marker CD31 (PECAM). Neither the surface area of CD31-positive structures, nor the absolute number of stained vessels differed significantly between both groups (p=0.111; Supplementary Figure 5A). Clinically, both patient groups showed no differences in lymph invasion or hemangiosis (not shown). The blood vessel structure did not differ between both groups, as evidenced by staining of smooth muscle actin, laminin and tenascin C (not shown). Interestingly, there were no significant differences in proliferation or apoptosis between both groups, as assessed by immunocytochemistry staining for Ki67 and cleaved caspase-3, respectively (Supplementary Figure 5B). However, patients with high CXC expression showed significantly increased expression of IFN_γ_, a main inducer of the CXCR3 ligands (p=0.0185, Supplementary Figure 5A). DNA microsatellite instability (MSI) has been associated with increased T-cell tumor infiltrates. The patients from the highest/lowest CXC quartiles were tested for microsatellite instability (38% MSI-high, 10 ouf of 26). However, there was no significant difference in the frequency of MSI-high cases between the CXC high vs. low expressing groups. No significant difference was observed regarding mean CXC expression, between patients with stable or unstable microsatellites (Supplementary Figure 5A).

### CXC-chemokine expression has anti-tumoral effect in mouse models

**Figure 5:**
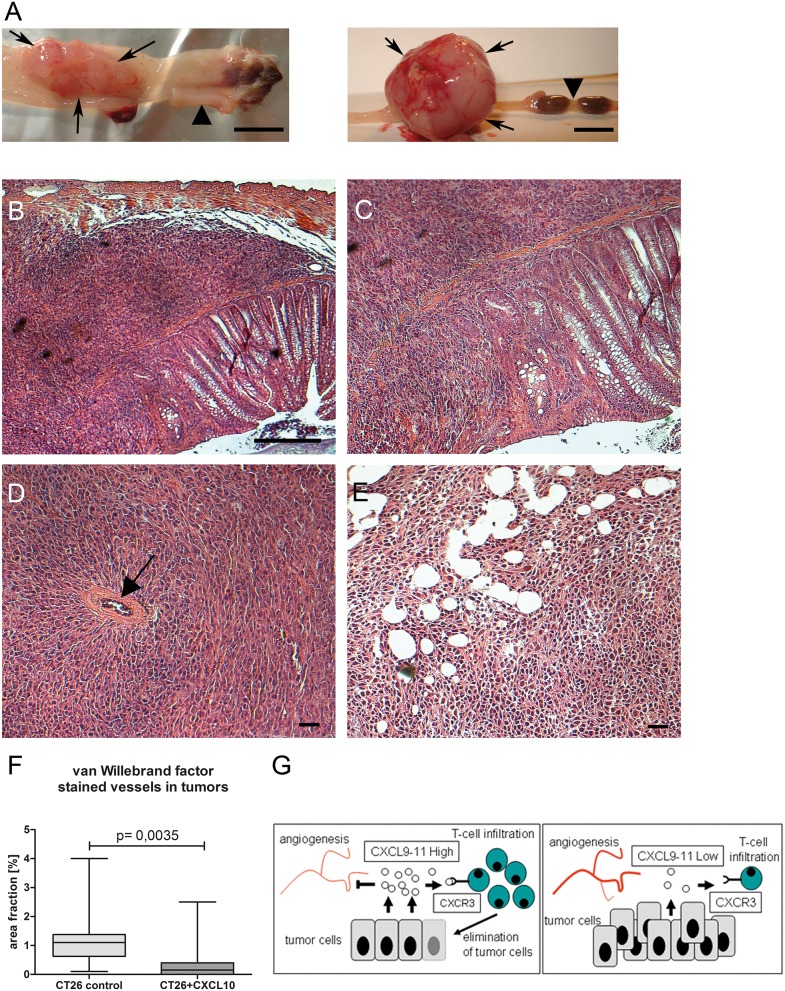
CXCL10-chemokine expression in orthotopic mouse model has anti-tumoral effect **(A)** Rectal tumors derived from orthotopically implanted control CT26 cells in immuno-competent isogenic host (left, rectum opened longitudinally), or in immuno-deficient host (right). Arrows denote tumor situs, arrowheads normal rectum. Size bar: 5 mm. **(B)** HE staining, poorly differentiated invasive adenocarcinoma, derived from CT26-control cells in isogenic host. Size bar: 50 μM. **(C)** Enlargement, spindle-shaped infiltration of muscularis. **(D)** Tumor derived from CT26-control cells in Rag1^-/-^ host, note vascularisation (arrow). **(E)** Tumor derived from CT26-CXCL10 cells in Rag1^-/-^ host, featuring necrosis. Size bar, 20 μM. **(F)** In immune-deficient Rag1-/- hosts, blood vessel density is significantly lower in tumors derived from CXCL10-expressing clones as compared to CT26 control clones. Cryosections were stained with anti-van-Willebrand-factor antibody for n=3 tumors each for both group, and ten high-power fields were quantified by ImageJ software. **(G)** Schematic summary on the role of CXCL9-11 in tumorigenesis. Left side: high intratumoral expression of CXCL9-11 inhibits blood vessel formation and attracts CTLs and T_H1_ cells, expressing the chemokine receptor CXCR3, leading to tumor regression. Right side: patients with low intratumoral chemokine expression lack beneficial T-cell infiltration, leading to unimpeded tumor growth and metastasis formation.

We investigated an assocation between CXC-chemokine expression and tumor aggressiveness in mouse models for colorectal cancer described earlier [21, 22]. Since the standard strain C57Bl/6 lacks CXCL11 expression [23]; CXCL11 was excluded from analysis. Intratumoral expression of CXCL9 and CXCL10 was significantly reduced in tumors from compound mutant RasApc mice, but not in single transgenic mice expressing oncogenic KRAS, which display lower tumor number and mortality (Figure 4D). Thus, chemokine expression was negatively correlated with the aggressivity of the tumor phenotype in the different genetic strains. Resected tumor explants were subjected to ex vivo culture and assessed by ELISA. Tumors from all genetic models showed significant chemokine secretion after cytokine stimulation (Supplementary Figure 6). To examine whether CXC-chemokines had a causal effect on tumorigenesis, an orthotopic mouse model was generated. CT26 murine rectal cancer cells were implanted in the rectum of isogenic, immune-competent hosts. CT26 cells express low endogenous levels of CXCL9 and CXCL10, even after cytokine stimulation, and no CXCL11 (Supplementary Figure 7A). Addition of exogenous murine CXCL10 had no discernible effects on CT26 cells, regarding proliferation or migration (Supplementary Figure 7B). In accordance, recombinant human CXCL10 failed to induce proliferation or cell migration in human HT29, CaCo2 and DLD1 cells (not shown). Thus, CT26 cells were engineered to express murine CXCL10. Neither cell proliferation nor migration was significantly altered in the stable clones (Supplementary Figure 7B). Expression of CXCL10 remained stable for 35 days, even after withdrawal of the selection antibiotic (Supplementary Figure 7C). Vector controls or CT26-CXCL10 cells (pools of three clones each), were implanted in immune-competent hosts, as well as in immune-deficient Rag1^-/-^ mice (Figure 5A, left and right panel, respectively), and tumor formation was monitored after 35 days. CT26-CXCL10 cells (expressing CXCL10) were unable to form tumors in immune-competent hosts, whereas control cells gave rise to poorly differentiated adenocarcinomas with high cellularity, extended areas of necrosis, and abundant mitoses (Figure 5B-5E). The anti-tumoral effect of CXCL10 expression was significant (p=0.0257, Chi-squared method; Table 2). Moreover, tumor cell invasion through all colonic layers was frequent in tumors derived from control CT26 cells (Figure 5C). Mesenteric and abdominal lymph node metastasis was observed in 14% of Rag1^-/-^ mice (4 out of 28), and in 4% of immune-competent hosts (1 out of 28), but only in tumors derived from control clones without CXCL10 expression (Table 2). Thus, tumors derived from CXCL10-expressing cells remained locally restricted and did not develop metastasis. The anti-tumoral effects of CXCL10 were mainly mediated by T- and B-cells, since CXCL10 expressing cells developed tumors in Rag1^-/-^ hosts, but not in immune-competent hosts. Tumor size, multiplicity and incidence were reduced in Rag1^-/-^ hosts implanted with CXCL10-expressing cells, as compared to control CT26 cells, but the differences did not attain significance (Table 2). Indeed, CT26-control tumors in immune-competent hosts were infiltrated by CD3-positive T-cells, as shown by immunohistochemistry (Supplementary Figure 8). In addition, blood vessel density was significantly reduced in tumors derived from CXCL10-expressing clones, as compared to tumors from control clones, as judged by staining against the endothelial marker van Willebrand factor on tissue sections (Figure 5F).

**Table 2:** Orthotopic tumor implantation in isogenic immune-competent and immune-deficient host

Host: wildtype	Tumor incidence	Mean size (mm^3^)	Metastasis incidence
**CT26-CXCL10**	0 / 19 (0%)	0	0 / 19 (0%)
**CT26-Control**	6 / 28 (21%)	454	1 / 28 (4%)
Fisher's exact test	^*^p=0.0351	/	n.s.*p*=0.5957
**Host: Rag1^-/-^(T /B cell-deficient)**	**Tumor incidence**	**Mean size (mm^3^)**	**Metastasis incidence**
**CT26-CXCL10**	2 / 20 (10%)	677	0 / 20 (0%)
**CT26-Control**	7 / 28 (25%)	2028	4 / 28 (14%)
Fisher's exact test	n.s.*p*=0.1753	n.s.*p*=0.4279, T-test	n.s.*p*=0.1052

Analysis of tumorigenesis in immune-proficient wildtype and Rag1^-/-^ hosts upon implantation of CT26-control cells, or CXCL10-secreting cells. No tumors were observable upon implantation of CXCL10-expressing cells in immunocompetent hosts, whereas control clones showed tumor formation in 18% of the animals. The difference in tumor indicence was significant. No significant difference was observable in Rag1-deficient host mice.

## DISCUSSION

The influence of adaptive immunity on colorectal cancer and other solid tumors is increasingly evident, and tumor infiltrating lymphocytes have remarkable prognostic power. However, it is still incompletely understood how T-cells are recruited into the tumor. Here, we propose a group of CXC-chemokines, soluble immune mediators, as key regulators for T-cell trafficking in colon carcinoma. We show that CXCR3-ligands, most notably CXCL11, constitute excellent independent prognostic biomarkers in a uni-centric retrospective patient collective, and we demonstrate anti-tumoral effects *in vivo* in a mouse model. However, for establishment of CXCL11 as a promising prognostic parameter in the clinic, further independent confirmation, based on large scaled and multicenter studies is still needed. We previously identified CXCL9-11, together with GZMB (Granzyme B), a key product of cytotoxic T-lymphocytes, as part of a prognostic gene expression signature in colorectal cancer [3, 4]. Here, we confirm these findings with an independent method, and within an independent patient collective. We report a tumor-specific differential expression of the CXC-chemokines and Granzyme B, with a remarkable degree of co-expression in individual patients. These findings are in accordance with earlier reports on up-regulation of CXCL9, CXCL10, CXCL11, and Granzyme B in colorectal carcinoma [24–29]. CXCL9 and CXCL10 were identified as T-cell homing factors in colon cancer [19, 30], and increased CXCL11 expression is a marker for less aggressive disease [10]. Interestingly, CXCL11 was the only transcript in our analysis that allowed significant distinction between cancer of all stages and normal mucosa. Moreover, CXCL11 was further down-regulated in liver metastases as compared to matched primary tumors, and above-threshold expression of CXCL9 and CXCL11 was significantly associated with longer cancer-specific and recurrence-free survival. Earlier studies reported a prognostic role for CXCL9 and CXCL10 expression in colon cancer [25, 30]. In the present study, CXCL11 remained the only independent prognostic parameter for post-operative survival upon multivariate analysis, even when adjusted to TNM staging. Variations in patient collectives are a likely explanation for the differences in prognostic power for the CXC-chemokines in individual studies. Our results establish intratumoral CXCL11 as promising prognostic parameter for colon cancer. Moreover, CXCL11 expression allowed stratification for distant recurrence risk in patients from UICC/AJCC stage II (Supplementary Table 3). This subgroup of patients is difficult to stratify for disease relapse by clinical standard methods [2, 31–34].

Next, we addressed the cellular origin of CXCL9-11 in colon cancer, which had been attributed previously to colon cancer cells and stroma cells [26, 35–39]. In accordance, all colon cancer cell lines tested secreted CXCL11 after stimulation with pro-inflammatory cytokines. Moreover, components of the tumor stroma, such as endothelial cells, pericytes and cancer-associated fibroblasts, were capable of CXCL11 expression. The pro-inflammatory cytokine IFN _γ_ is a known inducer of expression of CXCL9-11 [38]. This was confirmed on cell lines and on resected human and murine tumor explants. Of note, patient samples with high CXC-chemokine expression showed significantly increased transcript levels of IFN _γ_ Human tissue sections showed increased CXCL11 immunostaining in colon carcinoma as compared to adjacent normal mucosa, and the staining intensity was significantly correlated to the level of CXCL11 transcript. In accordance, tissue explants from colon carcinoma with above-threshold CXCL11 mRNA expression showed constitutive secretion of CXCL11, significantly increased compared to normal tissue.

The chemokines CXCL9-11 are angiostatic and serve as chemoattractants for T-cells and natural killer cells that express the receptor CXCR3 [8]. CXCR3 shows especially high levels on activated CD4^+^ T_H1_ and CD8^+^ cytotoxic effector T-cells, but is also reported to be expressed on NKT cells, regulatory-type cells such as T_regs_ and T_H17_ cells, endothelia, and at lower levels on T_H2_ cells [38, 40, 41]. CXCR3 ligands have been described to block angiogenesis and cause homing of T-cells into the tumor, leading to a preferential T_H1_–type recruitment and tumor growth inhibition [42, 43]. The density of T-lymphocytes has been proposed as prognostic indicator that outperforms the current clinical staging system [13, 14, 44–46]. The “immune contexture” is proposed as pivotal parameter for prognosis and survival in colon cancer [47]. Accordingly, a T_H1_-driven CD4+ population that supports formation of CD8^+^/GZMB^+^ effector cytotoxic cells has been associated with good prognosis [16]. In contrast, T_H2_–type cells and immune-suppressive FoxP3^+^ regulatory T-cells have been implicated in metastasis formation in colorectal cancer and other solid tumors [46, 48–51]. Given the strong association of CXC-chemokine expression with survival, we analyzed whether this positive effect could be mediated by T-cell infiltration. Indeed, CXCL11 expression was significantly correlated with the density of CD3^+^ T-cells and cytotoxic CD8^+^ effector T-cells, and highly significantly with CD4^+^ T_H1_ cells. In contrast, intratumoral populations of T_H2_, T_H17_ and immunosuppressive T_reg_ cells were unaffected by CXC-chemokine levels. In accordance, migration of patient-derived cytotoxic T-cells towards autologous colon cancer cells has been shown to be mediated by CXCR3 expressed on T-cells, and by CXCL11 expressed by tumor cells [24]. Along that line, high expression of CXCL9 and CXCL10, but not CXCL11, was associated with the attraction of memory CD8 T-cells with a specific TCR repertoire in colon cancer, indicative of good prognosis [30].

Thus, the excellent prognosis associated with high expression of CXCR3-ligands is likely to be mediated by CXCR3-dependent recruitment of a T_H1_-type anti-tumoral response (Figure 5F). However, it is still not understood why subgroups of colorectal cancer patients with differing “immune contextures” exist, either with or without beneficial inflammation. Our results indicate that CXCL11 expression allows a stratification between both groups. Several mechanisms, which are not mutually exclusive, may be the underlying cause: germline variations, e.g., polymorphisms in immune modulatory genes, somatic variations on genetic and epigenetic level within cancer cells, and lastly, differences in the intestinal microbiome. Together, these factors may either encourage or inhibit an efficient anti-tumoral immune response. Our results on tissue explants show that downregulation of CXC-chemokine expression, which we observed during tumor progression, is of a transient nature, and not likely the result of an irreversible loss-of-function.

Of note, an alternative function as autocrine pro-metastatic agents has been proposed for CXCR3-ligands, and reports on the functional contribution of CXC-chemokines to tumorigenesis are ambiguous [29]. Forced expression of CXCR3 on colon cancer cells promoted lymph node metastasis [52], and CXCL10 enhanced invasive and migratory capacities of colon cancer cells *in vitro* [20]. However, we failed to observe effects on cell migration by addition of CXCL10 in colorectal cancer cell lines of mouse or human origin. In order to provide evidence to solve this apparent conundrum, we investigated mouse models of colorectal cancer [21]. Since C57Bl/6-derived mice carry a natural null mutation for CXCL11 [23], only chemokines CXCL9 and CXCL10 could be studied. Both were significantly reduced in tumors from mouse models that spontaneously develop aggressive cancers. However, these data provide only correlative, and not causal evidence. Hence, mouse CT26 rectal cancer cells, which produce negligible amounts of CXCR3 ligands, were engineered to express murine CXCL10. Whereas no autocrine effects on proliferation or migration were observed, CXCL10-expressing CT26 cells were unable to form tumors in immune-proficient isogenic mice. In contrast, control cells induced invasive carcinoma. Thus, CXCL10 expression may lead to rapid tumor rejection by recruitment of CXCR3^+^ T-cells into the nascent tumor, in analogy to immune-competent mice implanted sub-cutaneously with CT26 cells, treated with an CXCL10-CXCL11 fusion chemokine [53]. The importance of adaptive immunity is underscored by our finding that cancer-cell derived CXCL10 did not protect from tumor formation in T- and B-cell deficient mice. However, tumorigenesis in immune-deficient hosts was partly reduced upon CXCL10 expression, which was attributable to decreased blood vessel density. Earlier reports showed lymphocyte-independent activity of CXCL10 in xenografts of human melanoma in immune-deficient hosts [54]. Importantly, we detected no metastasis in mice implanted with CXCL10-expressing clones. In fact, control of systemic spread could be a major contribution of CXC-chemokines to post-operative prognosis. Our findings are in good accordance with earlier results on CXCR3 and its ligands obtained in animal models, demonstrating anti-metastatic effects for colon cancer [55] or melanoma [56]. It has been reported that proliferation and apoptosis in primary colon cancer was not correlated to the expression of CXC-chemokines, nor to the density of intratumoral T cells [57]. Along that line, we observed no significant differences for proliferation and apoptosis of cancer cells between tumors with high or low CXCL11 expression. This suggests that adaptive immunity may not suffice for efficient control of the primary tumor. However, high intratumoral expression of CXCR3-ligands may encourage a lasting response that prevents metastasis formation. Our results establish the interferon-inducible CXC-chemokines as crucial mediators of tumorigenesis that initiate, exert and amplify profound effects on immune infiltration and tumor vasculature.

## MATERIALS AND METHODS

### Human samples

Tissue samples were obtained from 163 patients from our Surgical Department of the Klinikum rechts der Isar (Table 1). The study was approved by the local ethics committee (#1926/2007). Adjacent non-diseased colon mucosa from 28 patients was used as control. Tumors were classified according to the TNM system (7^th^ edition) by a pathologist. Only cases with completely resected (R0) tumors were included for prognosis assessment, and cases with R1, R2 and Rx status (total: n=43) were excluded from survival analysis (Supplementary Table 1).

### Cell culture

Human colorectal cancer lines HT29, HCT116, DLD1, SW480, CaCo2, and mouse CT26 cells [58] were cultured as described [59]. For culture of human brain vasculature pericytes (HBVP, #1200, ScienCell, Carlsbad, CA, USA), THP1 monocytes (American Type Culture Collection, Rockville, MD, ATCC), human umbilical vein endothelial cells (HUVEC; C-12203, PromoCell, Heidelberg, Germany), human cancer associated fibroblasts (CAF) [60, 61], see Supplementary Materials.

### Gene expression analysis

RNA was isolated from tissue samples based on histology-guided sample selection described earlier [59]. Briefly, ten frozen tissue sections were collected, haematoxylin/eosin staining was performed on each first and last section to ensure tumor cell content above 70% (verified by pathologist). RNA was isolated from intermediate slides using RNeasy Kit (Qiagen, Hilden, Germany), quantified, and checked by denaturing gel electrophoresis. Preparation of cDNA was performed using Fermentas RevertAid H-minus M-MulV Reverse Transcriptase (Fermentas/Fisher Scientific, Schwerte, Germany) and oligo-dT-T7 primer (Eurogentec, Cologne, Germany). Transcripts were determined by quantitative realtime reverse transcriptase–polymerase chain reaction (qRT-PCR) using the ABI PRISM 7300 system (Applied Biosystems, Foster City, CA, USA) with the dye SYBRGreen I, or the Roche Lightcycler 480 II (Roche, Penzberg, Germany). Expression of hypoxanthine-phosphoribosyl-transferase (HPRT) was used as internal reference; expression levels are indicated relative to the median expression in non-diseased colonic mucosa. Primer sequences: Supplementary Materials.

### Ex vivo culture of tumor explants

Tumors and histologically normal mucosa were dissected within 15 minutes after surgical resection by a pathologist (Supplementary Table 4). Three samples had to be excluded, because no neoplasm was detectable (2 cases), and one case was identified as metastatic lesion of gastric cancer. Samples were weighted and incubated at 37°C, 7% CO_2_ in DMEM (Invitrogen, Karlsruhe, Germany), with 1% Penicillin/Streptomycin (Biochrom, Berlin, Germany), 1% L-Glutamin (Biochrom) and 10% FCS (Biochrom), for 22h with or without addition of 10 ng/ml TNFα (Biosource) and 10 μg/ml IFN _γ_ (Invitrogen). ELISA was carried out with supernatants, normalized to tissue wet weight.

### Animal studies

Experiments on mice were performed in accordance with institutional and national guidelines and regulations. Macroscopic tumor analysis was carried out as previously described [62]. Wildtype mice and Rag1 deficient mice (Rag1^tm1Mom^) were maintained on BALB/c background (Charles River, Sulzfeld, Germany). Orthotopic cell implantation was performed based on a method described earlier [63]. Briefly, the colon descendens was prepared with a trypsin solution, washed with 5 ml HBSS (Gibco, Invitrogen, Karlsruhe, Germany), and isogenic cells were implanted (1×10^6^ CT26 cells, or clones CT26^+CXCL10^ or CT26^-CXCL10^). Total operating time was approximately 80min per mouse (30min incubation for trypsin and implanted cells, respectively). There was no mortality or tumor formation directly caused by the surgical procedure itself. Further details: Supplementary Materials.

### Statistics

Data analysis was done using Excel (Microsoft, Redmond, WA, USA) and GraphPad Prism (GraphPad, San Diego, CA, USA). Statistical analysis was performed with SPSS 16.0 (SPSS, Chicago, IL, USA) and the R system for statistical computing (www.R-project.org), including the add-on packages “survival” and “coin” [64]. Results were considered significant if *p*<0.05, correction for multiple testing was by Bonferroni-Holm. Multivariable Cox regression was performed to assess recurrence risk differences between derived subgroups in simultaneous consideration of potential confounding factors. Because of the relatively low number of critical events, multivariable regression analyses was performed consecutively (one-by-one inclusion of potential confounding factors) to avoid over-adjustment, as described in detail earlier [34]. Further details: see Supplementary Materials.

## SUPPLEMENTARY MATERIALS FIGURES AND TABLES


