# Supplementary material for: Interferon-inducible CXC-chemokines are crucial immune modulators and survival predictors in colorectal cancer
Source: Oncotarget. 2017 Sep 28;8(52):89998–90012. doi: 10.18632/oncotarget.21286 (PMC5685727; doi:10.18632/oncotarget.21286)
Supplement: Supplementary file 1 [file oncotarget-08-89998-s001.pdf]

Interferon-inducible CXC-chemokines are crucial immune modulators and survival predictors in colorectal cancer

SUPPLEMENTARY MATERIALS

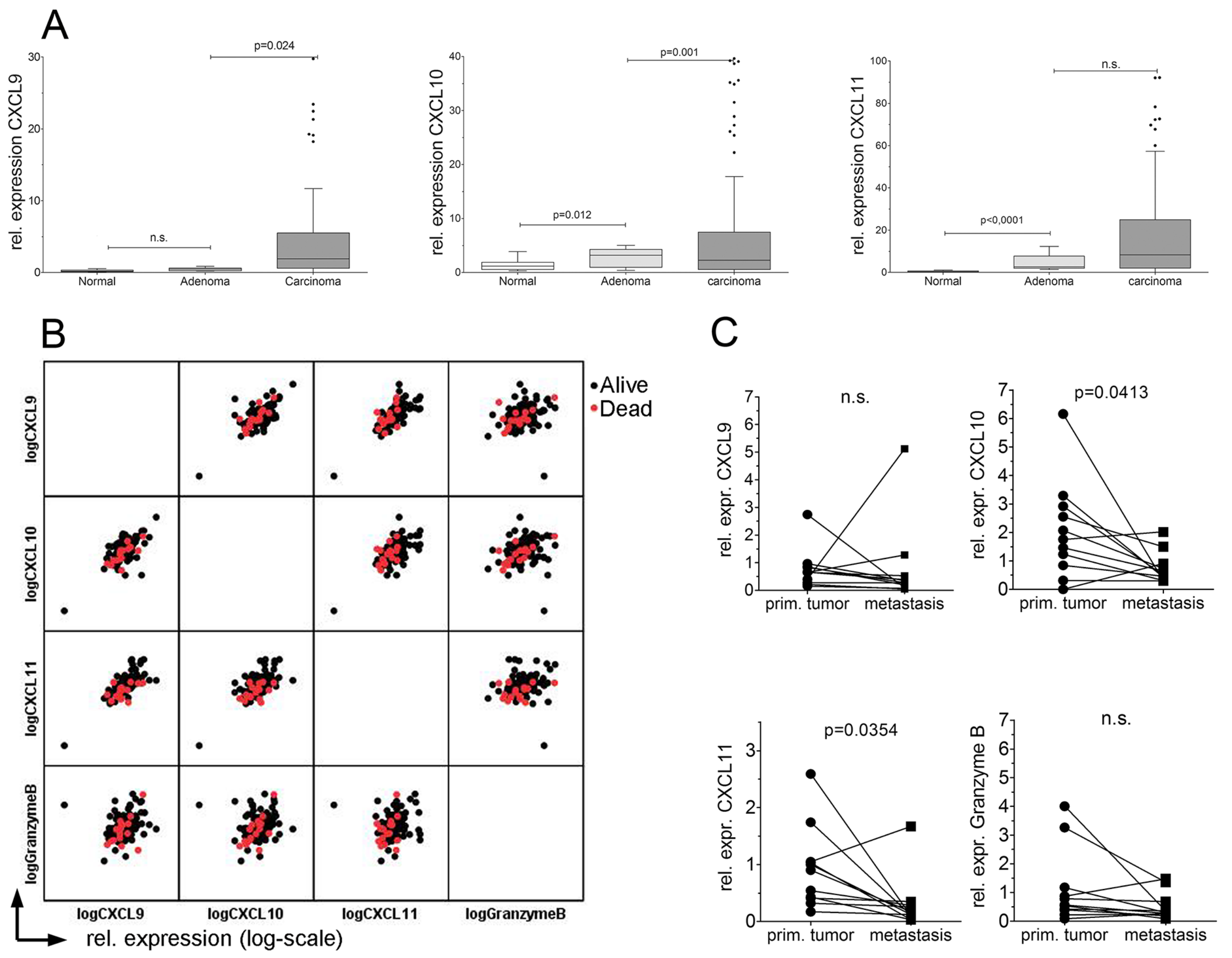

Supplementary Figure 1: (A) Chemokine mRNA expression in normal colon (n=28), benign adenoma (n=9), and carcinoma (n=163) by qRT-PCR, normalized to housekeeping gene HPRT. **(B)** Correlation matrix analysis shows strong degree of co-expression of chemokines, and negative correlation of expression with mortality (each dot represents mRNA expression value for one patient sample; red colour: tumor-related death, black colour: no event, patient alive during follow-up). **(C)** CXC-chemokine and granzyme B expression in matched primary colon tumors and liver metastasis from n=11 individual patients.

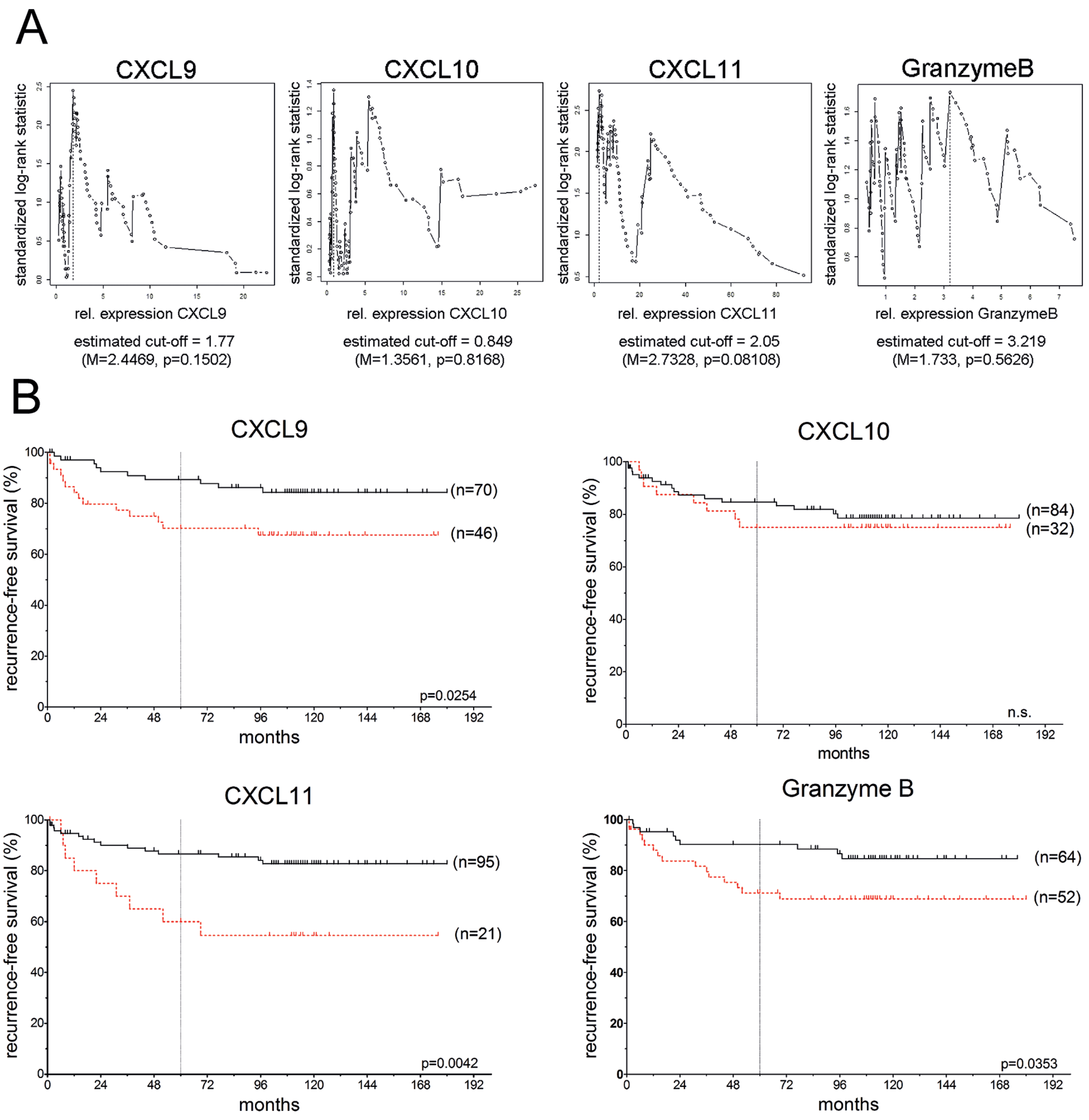

Supplementary Figure 2: (A) Cut-off determination for CXCL9, CXCL10, CXCL11, and granzyme B using maximally selected log-rank statistics. Time dependency of the events was considered for calculation. Estimated thresholds were: CXCL9 (1.77), CXCL10 (0.849), CXCL11 (2.05), and Granzyme B (3.219), expression relative to normal colon mucosa. **(B)** Kaplan-Meier Analysis of recurrence-free survival based on the above-mentioned thresholds for CXCL9, CXCL10, CXCL11 and Granzyme B.

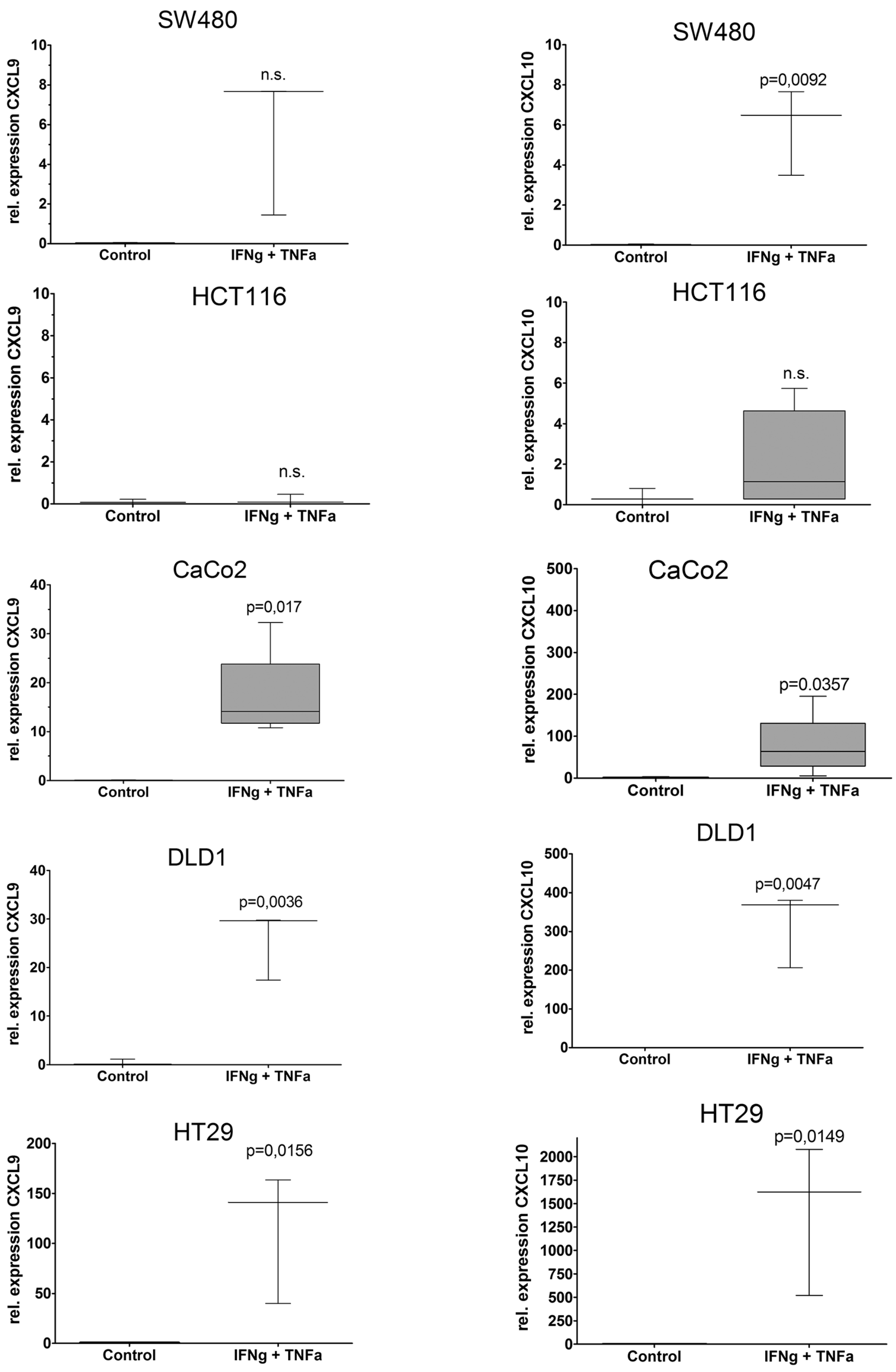

Supplementary Figure 3: CXCL9 (left column) and CXCL10 (right column) mRNA expression is induced by cytokine stimulation in human colorectal cancer cell lines (24h cytokine stimulation with TFNα+IFN $_{\gamma}$ in vitro).

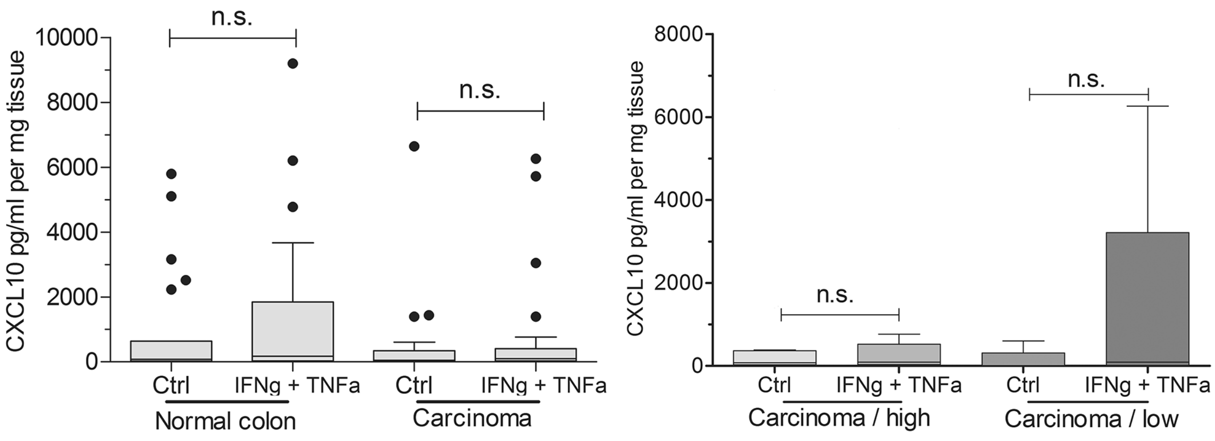

Supplementary Figure 4: Short term culture of human colon cancer explants and corresponding normal mucosa, CXCL10 ELISA on culture supernatants (n=22 patients; left panel). No significant differences were observed in CXCL10 secretion between tumor samples and normal mucosa. Next, based on CXCL10 mRNA expression levels, tumors were assigned to "high" expression (n=18), or "low" expression (n=7) group (right panel). CXCL10 protein secretion was not significantly different in both groups. Cytokine stimulation of initially "low" chemokine expressing tumors resulted in increased expression of CXCL10 (right panel), even though this difference did not attain significance.

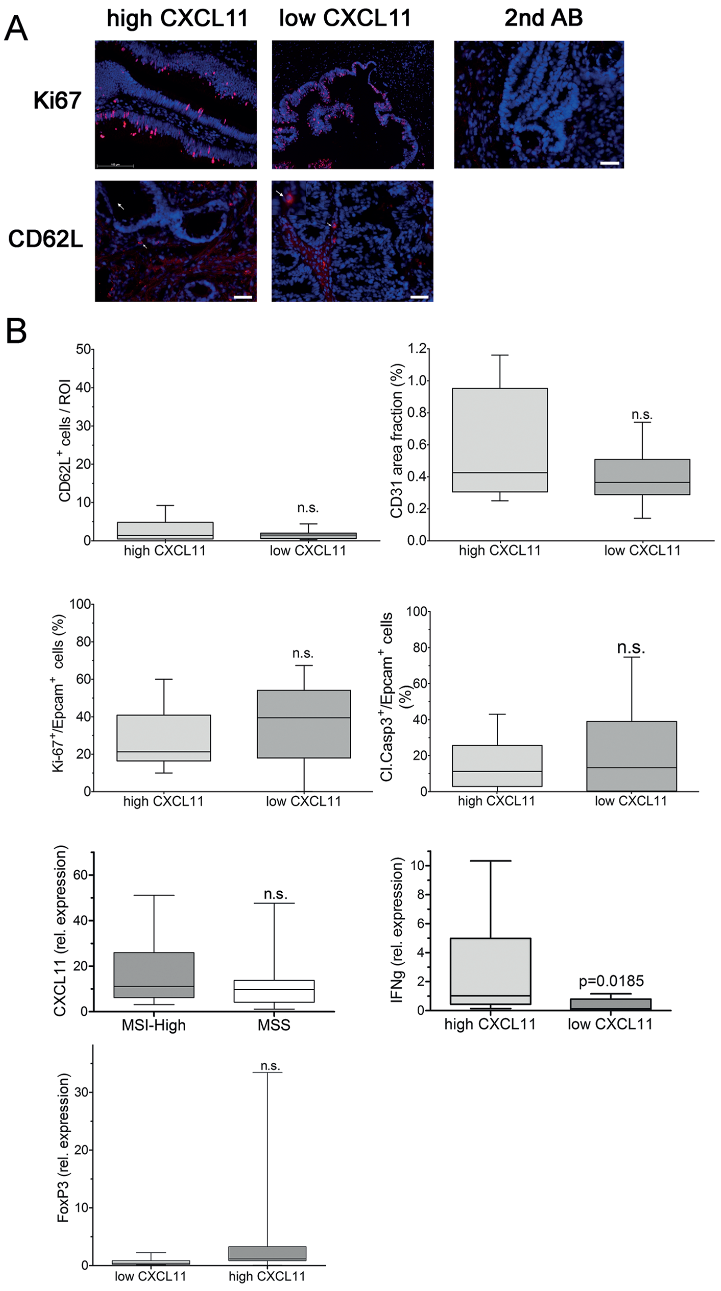

Supplementary Figure 5: (A) Quantification of CD62L-positive cells by immunofluorescence staining in tissue samples reveals no difference in the presence of naïve T-cells between CXCL11 high and low expressing tumors. Quantification of Blood vessel density (fraction of CD31-stained surface / ROI) is not significantly different between high and low CXCL11 expressing tumors. The number of proliferating tumor cells (Ki67-positive cells co-stained with the epithelial marker EpCam) is slightly lower in high CXCL11 expressing tumors, but not attaining significance. Number of tumor cells in apoptosis (quantified as percentage of cleaved-caspase3 positive cells of epithelial cells) is not significantly different. Intratumoral CXCL11 expression does not differ between patients with high microsatellite instability (MSI-High, n=10 patients) and stable microsatellite status (MSS, n=16 patients). Interferon-gamma (IFN $_{\gamma}$), the main inducer of CXCL11, is expressed at significantly higher levels in the CXCL11-high group (qRT-PCR). **(B)** Representative examples for the quantification of cell proliferation (anti-Ki67 staining, red); staining for L-selectin, a marker for naïve T-cells (CD62L, red), and secondary Antibody only as control for specificity (red). Nuclear counterstaining with DAPI (blue). Size bar: 50 µm.

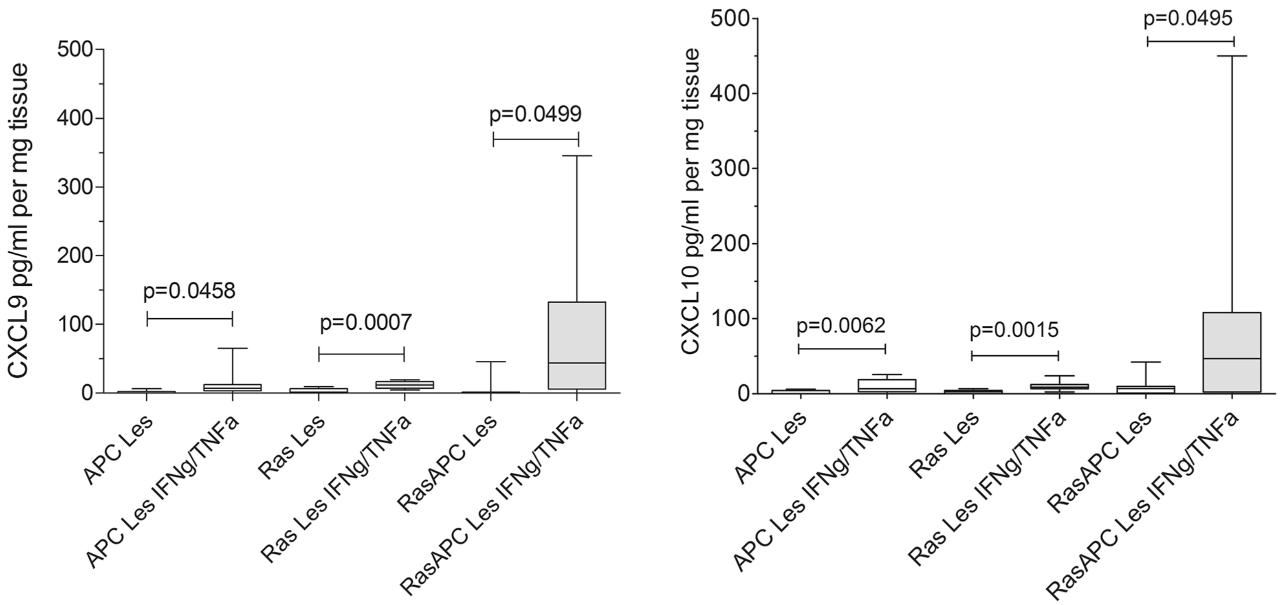

Supplementary Figure 6: Short term culture of intestinal cancer explants and corresponding normal mucosa from genetic mouse models. ELISA for CXCL9 and CXCL10 detection after short term culture (n>10 tumors per group). Secretion of CXCL9 and CXCL10 secretion was significantly higher after cytokines stimulation (murine IFN $_{\gamma}$ and TNFα), as compared to untreated controls, for each of the tested mouse models (APC, Apc$^{1638N/+}$, RAS, pvillin-Kras^{V12G}, combination of both alleles: RasAPC).

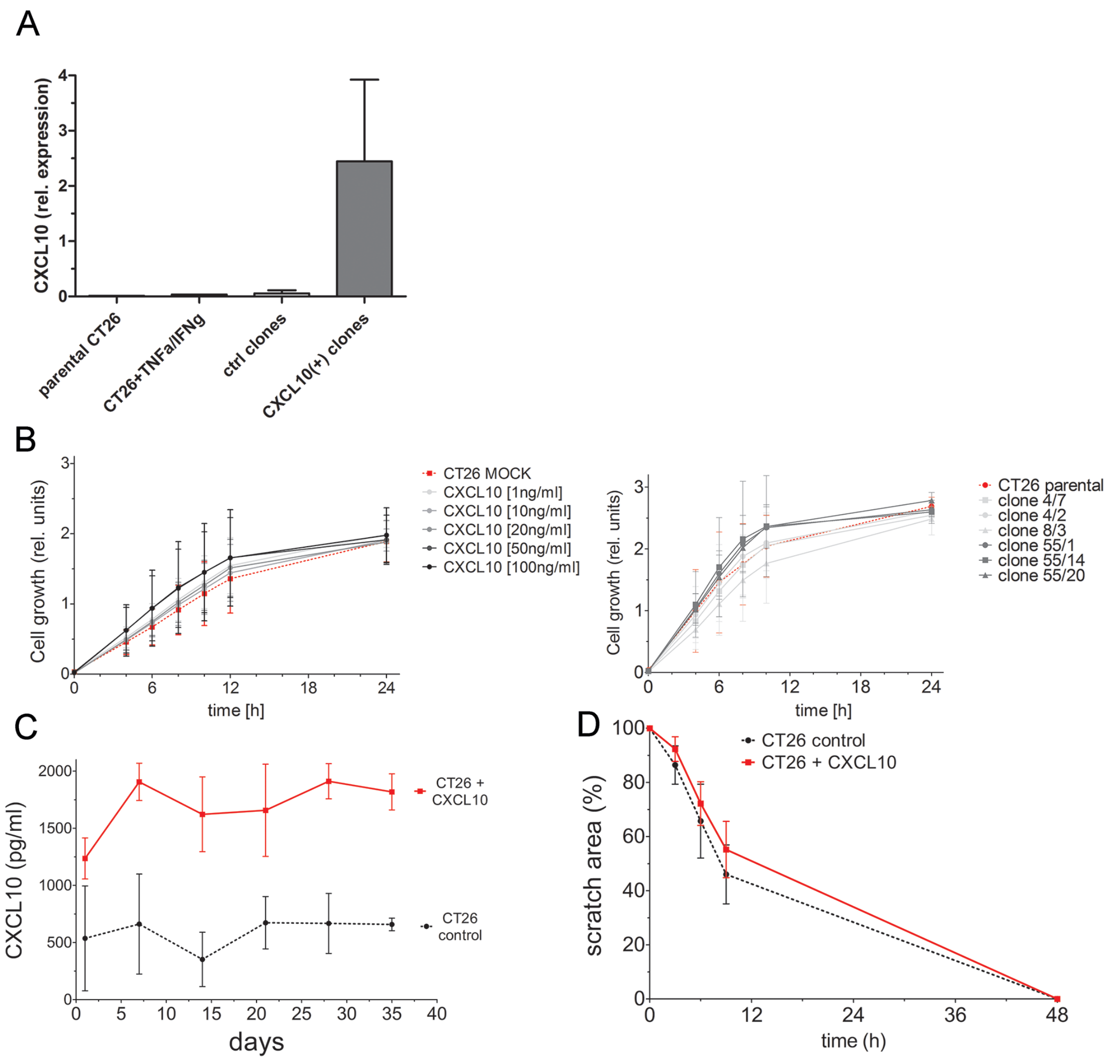

Supplementary Figure 7: (A) Expression of CXCL10 is barely detectable on mRNA in parental CT26 cells, even after cytokine stimulation (IFN $_{\gamma}$/TNFα). Three independent stable clones were generated that either express murine CXCL10, or the empty vector only. **(B)** Proliferation of mouse CT26 colon cancer cells is not significantly altered by addition of recombinant CXCL10, as evidenced by XTT proliferation assay (left; $3x10^4$ cells per well, mean ± SD of 3 assays carried out in duplicates). Accordingly, stable expression of CXCL10 in CT26 cells has no significant influence on cell proliferation, as evidenced by XTT proliferation assay on parental CT26 cells, three independent clones expressing CXCL10 (4/2, 4/7, 8/3), or three empty vector control clones (55/1, 55/14, 55/20) (right panel; $3x10^4$ cells per well, mean ± SD of 3 assays, carried out in duplicates). **(C)** Expression of CXCL10 remains stable for up to 35 days in culture without selection pressure (absence of antibiotic Zeocin). Vector control clones (mean of three clones) do not show significantly different CXCL10 levels, as compared to parental CT26 cells (400 pg/ml, not shown). ELISA carried out on cell culture supernatants, taken after indicated times of culture. Mean ± SD shown for three independent clones. **(D)** CT26 cell migration is not altered by addition of recombinant CXCL10. Standardized wound-healing assay (IBIDI-culture insert). Cells were treated with mitomycin-D to block cell proliferation before the assay. Mean ± SD for three experiments. Gap closure indicated as % of initial scratch area surface.

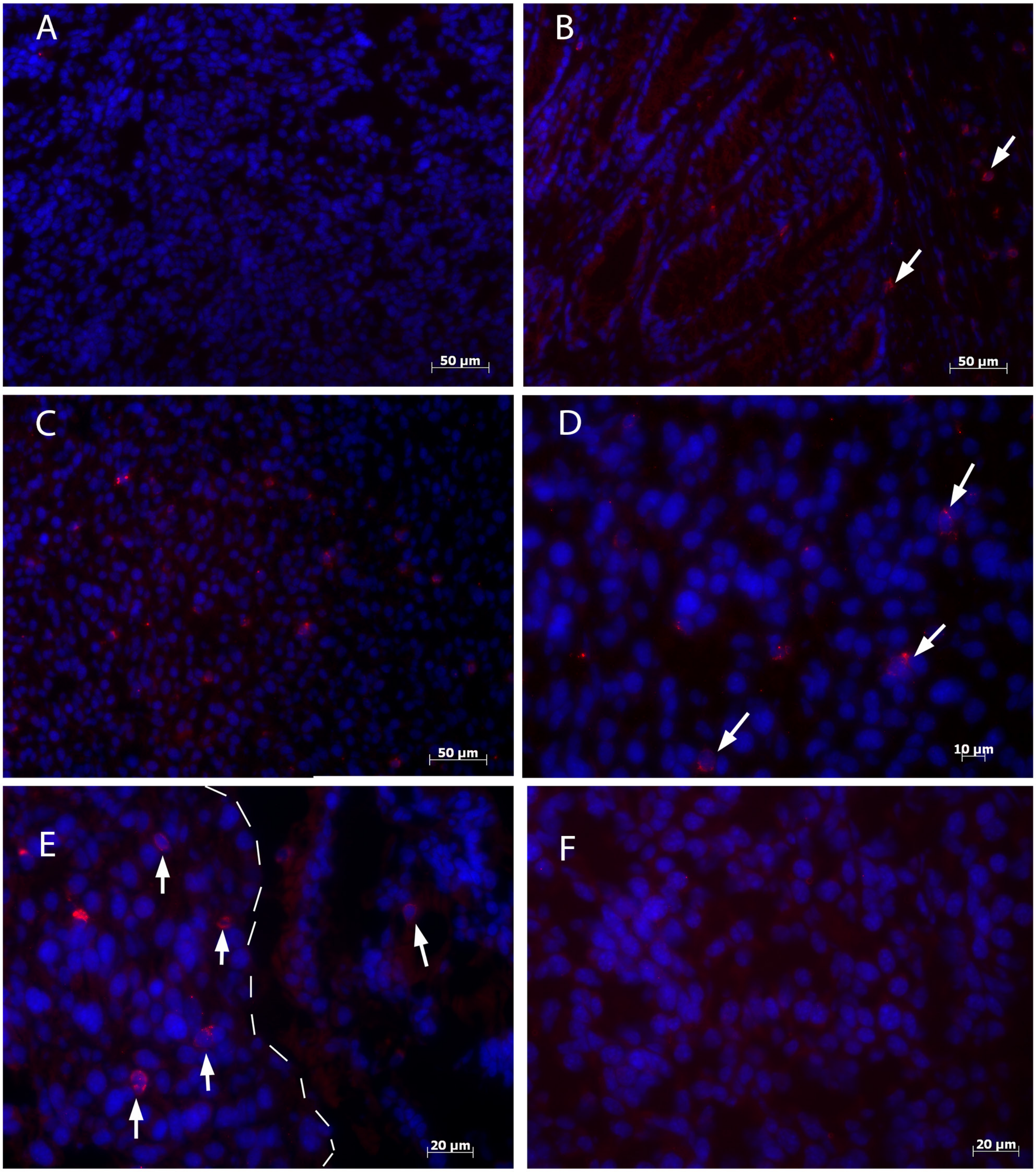

Supplementary Figure 8: CD3-positive T cells (stained with anti-CD3epsilon antibody, shown in red) infiltrate CT26-control tumors in immune-competent isogenic hosts. **(A)** Secondary antibody only on tumor section as staining control. **(B)** Normal colon tissue from wildtype mouse shows frequent CD3-positive cells (arrows). **(C)** Tumor-infiltrating CD3-positive cells in colon tumor from wildtype host, derived from a CT26-control clone. **(D)** Enlargement, positively stained T-cells marked by arrows. **(E)** Tumor infiltrating CD3-positive cells near invasive front of tumor (denoted by white line, CD3-positive cells marked by arrows), right side: adjacent normal colon. **(F)** As expected, no CD3-positive cells are detectable in lesions from immune-deficient Rag1$^{-/-}$ mice, formed by CT26-CXCL10 expressing cells.

Supplementary Table 1: Patient characteristics

Total n=163 [100%]	Male	Female
Sex (n[percent])	97 [59,51%]	66 [40,49%]
Age (mean +/- SD)	63,22 +/- 12,11 years	64,82 +/- 12,30 years
Age (range)	23-88 years	34-86 years
Tumor localization	[n]	percent
right	82	50.3
left	81	49.7
Tumor stage	[n]	percent
T1	3	1.8
T2	19	11.7
T3	107	65.6
T4	34	20.9
Grading	[n]	percent
G1	6	3.7
G2	104	63.8
G3	51	31.3
G4	2	1.2
UICC Stage	[n]	percent
Stage I	13	8.0
Stage II	75	46.1
Stage III	36	22.0
Stage IV	39	23.9.
Alive	[n]	percent
Yes	94	57.7
No	56	34.3
Tumor related death	39	23.9
missing data	13	8.0
Resection status	[n]	percent
complete resection (R0)	120	73.6
resection (R1,R2,Rx)	43	26.4
Recurrence	[n]	percent
No Recurrence	94	57.7
Recurrence (total)	33	20.3
missing data	36	22.0
Metastasis	[n]	percent
local recurrence only	2	1.2
distant Metastasis	47	28.8

Supplementary Table 2: CXC-chemokines are highly significantly co-expressed in individual tumor samples

		CXCL9	CXCL10	CXCL11	GranzymeB
**CXCL9**	coefficient of correlation		,619	,725	,367
	Sig. (two-sided)		<0,0001	<0,0001	<0,0001
	N		126	126	119
**CXCL10**	coefficient of correlation	,619		,552	,321
	Sig. (two-sided)	<0,0001		<0,0001	<0,0001
	N	126		126	119
**CXCL11**	coefficient of correlation	,725	,552		,288
	Sig. (two-sided)	<0,0001	<0,0001		,001
	N	126	126		120
**GranzymeB**	coefficient of correlation	,367	,321	,288	
	Sig. (two-sided)	<0,0001	<0,0001	,001	
	N	119	119	120	

Analysis by Spearman-Rho test, two-sided p-values are indicated.

Supplementary Table 3: Characteristics of patient subset with complete resection of primary tumor (R0), n=120

Total collective n=120 [100%]	Male	Female
**Sex (n[percent])**	76 [63,33%]	44 [36,67%]
**Age (mean +/- SD)**	63,7 +/- 11,8 years	66,5 +/- 10,58 years
**Age (range)**	23-88 years	44-86 years
**Tumor localization**	**[n]**	**percent**
right	65	54,17
left	55	45,83
**Tumor stage**	**[n]**	**percent**
T1	3	2,50
T2	17	14,17
T3	79	65,83
T4	21	17,50
**Grading**	**[n]**	**percent**
G1	5	4,17
G2	79	65,83
G3	34	28,33
G4	2	1,67
**UICC Stage**	**[n]**	**percent**
Stage I	13	10,83
Stage II	74	61,67
Stage III	26	21,67
Stage IV	7	5,83
**Alive**	**[n]**	**percent**
Yes	86	71,67
No	34	28,33
Tumor related death	17	14,17
**Resection status**	**[n]**	**percent**
complete resection (R0)	120	100,00
resection (R1,R2,Rx)	0	0,00
**Recurrence**	**[n]**	**percent**
No Recurrence	96	80,00
Recurrence (total)	24	20,00
**Metastasis**	**[n]**	**percent**
local recurrence only	2	1,67
distant Metastasis	22	18,33

Supplementary Table 4: Association of CXCL11 expression with distant recurrence risk in stage II colon cancer

UICC stage II patients only	Disease relapse	No relapse
high CXCL11 expression	10	53
low CXCL11 expression	4	4

p=0,0431 (Fisher's exact test)

Supplementary Table 5: patient characteristics for samples used for ex vivo organ culture (surgical specimen of freshly resected colorectal cancer)

Total collective n=22 [100%]	Male	Female
**Sex (n[percent])**	12 [54%]	10 [46%]
**Age (mean +/- SD)**	74 +/- 19 years	67 +/- 9 years
**Age (range)**	35-89 years	47-84 years
**Tumor localization**	**[n]**	**percent**
right	10	45.5%
left	10	45.5%
rectum*	2	9.0%
**UICC Stage**	**[n]**	**percent**
Stage I	2	9.0
Stage II	6	27%
Stage III	9	41%
Stage IV	5	23%

*no pre-treatement prior to surgical resection
